# Características sociodemográficas y clínicas relacionadas con la condición final de pacientes intoxicados por paraquat en un hospital del suroccidente de Colombia

**DOI:** 10.7705/biomedica.6361

**Published:** 2022-09-02

**Authors:** Yalila Andrea Ordóñez-Zarama, Daniel Jurado-Fajardo, María Camila Paredes- Panesso, David Alejandro Rosero-Bello, Franco Andrés Montenegro-Coral, José Alirio Risueño-Blanco

**Affiliations:** 1 Servicio de Toxicología, Hospital Universitario Departamental de Nariño, Pasto, Colombia Hospital Universitario Departamental de Nariño Pasto Colombia; 2 Programa de Medicina, Universidad de Nariño, Pasto, Colombia Universidad de Nariño Universidad de Nariño Pasto Colombia; 3 Grupo GIISE, Programa de Medicina, Universidad Cooperativa de Colombia, Pasto, Colombia Universidad Cooperativa de Colombia Universidad Cooperativa de Colombia Pasto Colombia; 4 Departamento de Salud Pública, Programa de Epidemiología, Universidad de Caldas, Manizales, Colombia Universidad de Caldas Universidad de Caldas Manizales Colombia

**Keywords:** paraquat, plaguicidas, herbicidas, envenenamiento, creatinina, mortalidad, Paraquat, pesticides, herbicides, poisoning, creatinine, mortality

## Abstract

**Introducción.:**

El herbicida paraquat constituye la primera causa de decesos por intoxicaciones en distintos países.

**Objetivo.:**

Relacionar las características sociodemográficas y clínicas con la condición final de pacientes intoxicados por paraquat atendidos en un hospital del suroccidente de Colombia.

**Materiales y métodos.:**

En este estudio observacional, descriptivo, analítico, transversal y retrospectivo, se revisaron las historias clínicas de pacientes atendidos por intoxicación con paraquat en una institución de tercer nivel de complejidad en Pasto (Colombia) entre el 2013 y el 2018. Se recolectó la información sobre la condición final (vivo o fallecido) de cada paciente, así como los datos sociodemográficos, clínicos y de los exámenes paraclínicos. Se establecieron comparaciones por grupos y se diseñó un modelo de regresión logística binaria.

**Resultados.:**

Se filtró la información de 299 registros y se analizaron finalmente 160 casos. Las características relacionadas con la condición final de los pacientes fueron el tiempo de estancia (OR=0,124; IC_95%_ 0,03-0,6; p=0,009), la frecuencia cardiaca (OR=35,65; IC_95%_ 1,44-884,78; p=0,029) y la creatinina inicial (OR=1,73; IC_95%_ 1,23-2,44; p=0,002).

**Conclusiones.:**

La proporción de la letalidad fue mayor entre los pacientes con estancia hospitalaria corta, taquicardia y elevación de la concentración sérica de creatinina al ingreso. Este reporte puede ser útil como fundamento de una escala de gravedad para detectar a los pacientes con resultados adversos en la fase temprana para que puedan recibir una intervención oportuna.

## Abstract (en)

**Introduction:**

Paraquat is an agrochemical that constitutes the first cause of death by poisoning in different countries.

**Objective:**

To relate sociodemographic and clinical characteristics with the final condition of patients intoxicated by paraquat in a hospital in southwestern Colombia.

**Materials and methods:**

This was an observational, descriptive, analytical, crosssectional, and retrospective study. We reviewed the medical records of patients diagnosed with paraquat poisoning at a level III hospital in Pasto, Colombia, from 2013 to 2018.

We collected the data regarding their final condition (alive or deceased) and their sociodemographic, clinical, and paraclinical information. We established group comparisons and designed a binary logistic regression model.

**Results:**

We reviewed 299 records and, after the exclusion, we analyzed 160 cases. The characteristics related to the final condition of the patients were length of stay (OR = 0.124; 95% CI: 0.03-0.6; p = 0.009), heart rate (OR = 35.65; 95% CI: 1.44-884.78; p = 0.029), and initial creatinine (OR = 1.73; 95% CI: 1.23-2.44; p = 0.002).

**Conclusion:**

The proportion of case fatality was higher in patients with short hospital stay times and elevated heart rates and admission creatinine levels. This report may be useful as a rationale for the creation of a severity scale for the early detection of patients with adverse outcomes and their timely treatment.

Según la Organización Mundial de la Salud (OMS), la intoxicación por plaguicidas constituye un verdadero problema de salud pública en el mundo, especialmente en países en desarrollo, donde se presenta la mayor cantidad de casos ^1^^,^^2^. Se estima que a nivel global ocurren tres millones de intoxicaciones ^1^^,^^3^, algunas de tipo accidental, asociadas con labores agrícolas, y otras con intención suicida ^2^^,^^4^^,^^5^; la mortalidad global es de 250.000 a 370.000 personas al año; el 90 % de estas muertes se debe al uso de la sustancia con fines suicidas ^6^. Entre los plaguicidas responsables, el paraquat es uno de los agentes con mayor índice de intoxicación y constituye la primera causa de decesos en distintos países, con tasas de mortalidad que varían entre regiones: en Estados Unidos se ha reportado una letalidad del 54 %; en Francia, del 74 %; en Irán, entre el 43 y el 55,2 %; y en las poblaciones china y coreana, entre el 42,2 y el 88,3 % ^7^^-^^10^.

Según el Instituto Nacional de Salud y el Sistema Nacional de Vigilancia en Salud Pública (Sivigila), en el 2017 se reportaron en Colombia 209.823 casos de intoxicación por sustancias químicas, de los cuales 67.499 (32.2 %) fueron por plaguicidas y el 43,5 % con intención suicida ^11^. Se considera, además, que hay subestimación de los casos porque la carga global de la enfermedad no se ha determinado completamente y hay subregistro en algunos lugares del país, por lo que el impacto económico y en salud puede ser mayor ^12^^,^^13^. En los departamentos de Nariño y Putumayo, cuya actividad económica se fundamenta en la agricultura, el uso de diversos productos agrícolas y plaguicidas con elevado potencial tóxico para las personas es extendido ^14^ y, según el reporte del Instituto Nacional de Salud, la frecuencia de mortalidad por sustancias químicas allí es alta ^13^.

El paraquat (dicloruro de N, N’-dimetil-4,4’-bipiridinio), también conocido como dicloruro de paraquat, es un herbicida empleado para el control de malezas de uso extendido en la agricultura que tiene efecto tóxico para los humanos, por lo que la OMS lo ha clasificado en la categoría toxicológica II, es decir, moderadamente peligroso ^14^, en tanto que la *Environmental Protection Agency* (EPA) de los Estados Unidos lo cataloga como de uso restringido bajo licencia ^15^. Entre las características toxicocinéticas más importantes del paraquat, se destacan la de ser un herbicida orgánico e hidrosoluble que actúa por contacto y presenta un volumen de distribución en el organismo que oscila entre 1 y 2,75 L/kg, por lo que se lo considera liposoluble, o sea que accede a los tejidos periféricos con facilidad ^15^^,^^16^. La intoxicación por paraquat produce complicaciones agudas (hepáticas, renales y pulmonares, entre otras) que conducen a la muerte ^16^.

En cuanto a la cinética de distribución del paraquat, el proceso se da un modelo de tres compartimentos: el de distribución (plasma), el de captación y eliminación rápida (riñón), y el de absorción lenta (pulmón) ^17^^,^^18^. Se absorbe de manera rápida e incompleta y se excreta en la orina entre 12 y 24 horas después de ingerido ^19^. El tóxico ingresa al pulmón, su órgano blanco, en concentraciones 10 a 15 veces superiores a las del plasma ^20^; en dosis altas, también puede ocasionar falla multiorgánica al lesionar otros órganos importantes, como corazón, riñones, hígado, glándulas suprarrenales o bazo, el sistema nervioso central o los músculos ^8^. Las manifestaciones clínicas de la intoxicación se inscriben en tres categorías de gravedad: leve, inicialmente con lesiones en la cavidad bucal y manifestaciones gastrointestinales; moderada, con insuficiencia renal aguda, y grave, cuando produce hepatitis aguda, neumonía y fibrosis pulmonar que desemboca en la muerte dos a tres semanas después de la exposición ^8^.

Los tratamientos más utilizados en la intoxicación por paraquat son los adsorbentes gástricos, la filtración extracorpórea y el manejo farmacológico con antioxidantes, antiinflamatorios e inmunosupresores ^21^. Se hace seguimiento clínico y de los marcadores bioquímicos para determinar la reacción terapéutica del paciente y los criterios pronósticos de supervivencia ^22^^-^^24^. No existen antagonistas farmacológicos ni agentes quelantes contra el paraquat ^17^, aunque la 5-hidroxi-1-metilhidantoína (HMH) podría reducir la injuria pulmonar ^25^. Actualmente, no se cuenta con un antídoto específico que realmente repercuta en el pronóstico del paciente. Otros factores asociados con la intoxicación por paraquat, descritos como predictores de mal pronóstico, son cantidad ingerida, valor sérico de la creatinina, hiperamilasemia, hiperglucemia y compromiso multiorgánico ^26^^-^^30^.

Ante el incremento de pacientes intoxicados con paraquat provenientes de los departamentos de Nariño y Putumayo admitidos en un hospital de tercer nivel de complejidad de referencia en el suroccidente colombiano, y dada la escasez de estudios sobre la intoxicación aguda por este herbicida en Nariño, se decidió hacer el presente estudio para determinar relacionar entre las características sociodemográficas y clínicas, y el resultado final de los pacientes intoxicados con paraquat.

## Materiales y métodos

Se hizo un estudio de tipo observacional, descriptivo de fase analítica, analítico, transversal y retrospectivo mediante la revisión de las historias clínicas de pacientes que ingresaron a un hospital de tercer nivel de complejidad en Pasto, durante el periodo 2013-2018 y con diagnóstico de intoxicación por paraquat según el CIE-10 (T603, T608, T609). Se excluyeron registros con información de identificación incompleta de los pacientes o con intoxicaciones por otras sustancias. Se recopiló la información sobre la condición final (vivo o fallecido) de cada paciente y su información sociodemográfica, clínica y de exámenes paraclínicos. La gravedad de la intoxicación se clasificó según la guía para el manejo de emergencias toxicológicas del Instituto Nacional de Salud vigente en la fecha del estudio, como: leve, con ingestión menor de 20 mg/kg y paciente asintomático o con vómito, diarrea o efecto corrosivo; moderada, con ingestión entre 20 y 40 mg/ kg, o paciente con síntomas de intoxicación leve y, además, con toxicidad sistémica más fibrosis pulmonar; y grave, con ingestión mayor de 40 mg/kg, perforación del tubo digestivo o falla orgánica multisistémica ^31^.

Los exámenes complementarios iniciales se tomaron en el día uno y, los finales, entre el segundo y quinto días (en caso de registros consecutivos incompletos, se calculó el promedio de los valores); los datos de las demás variables se extrajeron del registro del día de ingreso.

Se utilizó el test de Kolmogórov -Smirnov con todas las variables cuantitativas para establecer si había distribución normal o no; en los casos de distribución normal, estas se reportaron como medias y desviación estándar, y en caso contrario, como medianas y rango intercuartílico.

Las variables cualitativas se reportaron con sus respectivas frecuencias y porcentajes. Las relaciones, medidas con odds ratio (OR), de la variable dependiente (condición final) con variables categóricas se analizaron con pruebas de ji al cuadrado de homogeneidad y con variables cuantitativas mediante U de Mann Whitney (sin distribución normal; en caso de encontrar diferencias se consideró aplicar la prueba de la mediana o en su defecto categorizarlas con criterios teóricos y de su distribución) o t de Student para muestras independientes (con distribución de Gauss, previa verificación de homocedasticidad con test de Levene y en caso de heterocedasticidad se emplea test de Welch). Para controlar la confusión, se hizo un análisis multivariado con regresión logística binaria en el que solamente se incluyeron aquellas variables con una pérdida de datos inferior al 20 %. Se establecieron intervalos de confianza (IC) del 95 % y un nivel de significación de 5 %; los análisis se hicieron en el programa *Statistical Package for Social Science™*, versión 25 (Chicago, IL, USA; licencia de la Universidad Cooperativa de Colombia). El diagrama de caja y bigotes fue diseñado en el lenguaje R, versión 4.1.1.

Según el artículo 11 de la resolución 8430 de 1993 del Ministerio de Salud y Protección Social de Colombia, este estudio se clasifica como una investigación sin riesgo, pues únicamente se revisaron las historias clínicas. Fue aprobado por el Comité de Investigaciones y Bioética del Hospital Universitario Departamental de Nariño y por el Comité de Investigación de la Facultad de Medicina de la Universidad Cooperativa de Colombia en Pasto.

## Resultados

**Figura 1:**
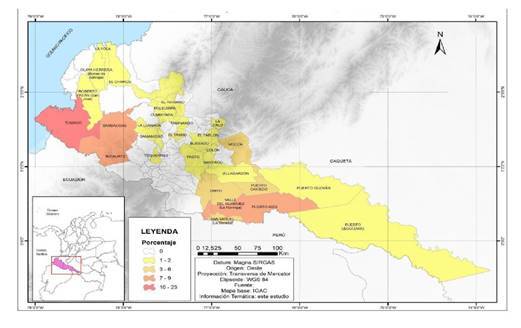
Casos de intoxicación por paraquat atendidos en el hospital de tercer nivel, departamentos de Nariño y Putumayo, Colombia, 2013-2018

Se recolectó la información de 299 registros y, después de la exclusión, se analizaron 160 casos. Las características sociodemográficas, clínicas y complementarias de los pacientes, se presentan en el cuadro 1, y su relación con la condición final de los pacientes, así como el análisis multivariado, se resumen en el cuadro 2. En la figura 1, se presentan los lugares de procedencia de los pacientes y un resumen de la proporción de casos de intoxicación con paraquat por municipio.

**Cuadro 1:** Características sociodemográficas, clínicas y complementarias de los pacientes intoxicados con paraquat. Departamentos de Nariño y Putumayo, Colombia, 2013- 2018

Característica		Categorías	n (%)	Condición final n (%)	
				Vivo	Muerto
Sociodemográficas					
	Sexo	Masculino	89 (55,6	68 (77,3)	20 (22,7)
		Femenino	71 (44,4)	53 (74,6)	18 (25,4)
	Departamento	Nariño	88 (55)	67 (77)	20 (23)
		Putumayo	72 (45)	54 (75)	18 (25)
	Procedencia	Rural	132 (82,5)	101 (77,1)	30 (22,9)
		Urbano	28 (17,5)	20 (71,4)	8 (26,8)
	Escolaridad	Con estudios	82 (51,25)	70 (85,4)	12 (14,6)
		Otros	78 (48,75)	51 (66,2)	26 (33,8)
	Ocupación	Desconocido	63 (39,4)	38 (61,3)	24 (38,7)
		Agricultor	41 (25,6)	34 (82,9)	7 (17,1)
		Ama de casa	27 (16,9)	27 (100)	0 (0)
		Otros	29 (18,1)	22 (75,9)	7 (24,1)
	Régimen de salud	Subsidiado	133 (83,1)	100 (75,8)	32 (24,2)
		No subsidiado	27 (16,9)	21 (77,8)	6 (22,2)
Clínicas cualitativas					
	Cantidad ingerida (gravedad)	Moderada	124 (77,5)	99 (80,5)	24 (19,5)
		Grave	36 (22,5)	22 (61,1)	14 (38,9)
	Compromiso	Digestivo	58 (36,25)	51 (87,9)	7 (12,1)
		Sin compromiso	52 (32,5)	49 (96,1)	2 (3,9)
		Renal	27 (16,88)	13 (48,1)	14 (51,9)
		Otros	23 (14,37)	8 (34,8)	15 (65,2)
	Falla renal	Sin falla renal	106 (66,3)	93 (88,6)	12 (11,4)
		Con falla renal	54 (33,7)	28 (51,9)	26 (48,1)
	Diálisis	No	142 (88,8)	114 (80,9)	27 (19,1)
		Sí	18 (11,2)	7 (38,9)	11 (61,1)
	Vía de exposición	Oral	152 (95)	113 (74,8)	38 (25,2)
		Otra	8 (5)	8 (100)	0 (0)
	Causa	Intencional	141 (88,1)	108 (77,1)	32 (22,9)
		No intencional	19 (11,9)	13 (68,4)	6 (31,6)
	Tiempo entre ingestión y atención médica	Mayor de 24 horas	104 (65 %)	81 (77,9)	23 (22,1)
		Menor de 24 horas	56 (35 %)	40 (27,7)	15 (27,3)
	Tiempo de estancia hospitalaria (categorizada)	1 a 5 días	55 (34,4 )	37 (67,3)	18 (32,7)
		6 a 10 días	66 (41,2)	52 (78,8)	14 (21,2)
		11 días o más	38 (23,8)	32 (84,2)	6 (15,8)
		Sin dato	1 (0,6)	-	-
	Frecuencia cardíaca (categorizada)	Menor de 60 lpm**	4 (2,5)	3 (100)	0 (0)
		Entre 60 y 100 lpm**	138 (86,2)	111 (80,4)	27 (19,6)
		Mayor de 100 lpm**	18 (11,3)	7 (38,9 %)	11 (61,1)
	Glóbulos blancos (valor inicial)	0 a 11.000 por mm3	50 (31,25)	44 (88)	6 (12)
		Más de 11.000 a 16.000 por mm^3^	44 (27,5)	36 (81,8 )	8 (18,2)
		Más de 16.000 a 20.000 por mm^3^	22 (13,75)	16 (72,7)	6 (27,3)
		Más de 20.000 por mm^3^	14 (8,75)	6 (42,9)	8 (57,1)
		Sin dato	30 (18,75)	-	-
Característica		Medida de tendencia central (dispersión)*	Porcentaje de datos perdidos (n)	Condición final (medida de tendencia central - dispersión-)	
				Vivo	Muerto
Clínicas cuantitativas					
	Tiempo de estancia hospitalaria	7 días (RIC 6)	0,63 % (1)	7 días (RIC 6)	6 (RIC: 5,25)
	TA** sistólica	118 mm Hg (RIC 19,25)	1,25 % (2)	118 (RIC: 18)	120 (RIC: 25,5)
	TA** diastólica	70 mm Hg (RIC 15)	1,25 % (2)	70 (RIC: 10)	70 (RIC: 20)
	TA** media	85,3 mm Hg (RIC 13,33)	1,25 % (2)	85,3 (RIC: 12,3)	85 (RIC: 17,5)
	Temperatura	36,2 °C (RIC 0,5)	0,63 % (1)	36,3 (RIC: 0,5)	36,1 (RIC: 0,7)
	Frecuencia cardíaca	80 lpm** (RIC 4)	0 (0)	80 (RIC: 13)	81,5 (RIC: 30,25)
	Frecuencia respiratoria	18 rpm** (RIC 2)	0 (0)	18 (RIC: 2)	16 (RIC: 4)
	Hemoglobina inicial	14,11 mg/dl (DE=1,91)	18,75 % (30)	14,14 (DE=1,92)	14 (DE=1,9)
	Hemoglobina final	13,35 mg/dl (DE=1,86)	40 % (64)	13,33 (DE=1,78)	13,45 (DE=2,28)
	Hematocrito inicial	41,91 % (DE 5,21)	18,75 % (30)	42 (DE=5,17)	41,55 (DE=5,46)
	Hematocrito final	39,62 (DE=5,31)	40 % (64)	39,6 (DE=5,08)	39,71 (DE=6,54)
	Glóbulos blancos (valor inicial)	12.600 por mm3 (RIC=7400)	18,75 % (30)	12.300 (RIC: 6725)	16.050 (RIC: 12225)
	Glóbulos blancos (valor final)	10.700 por mm3 (RIC=5060)	40 % (64)	9.875 (RIC: 4588)	13.650 (RIC: 7214)
	Diferencial de neutrófilos inicial	82,1 % (RIC=16,25)	19,4 % (31)	80 (RIC: 15,9)	89,6 (RIC: 9,9)
	Diferencial de neutrófilos final	71,1% (RIC=24,68)	40 % (64)	66,95 (RIC: 23,1)	84,9 (RIC: 11,2)
	Plaquetas (valor inicial)	278.346,2 por mm3 (DE=70.973,38)	18,75 % (30)	276.863 (DE=69963)	283.750 (DE=75616)
	Plaquetas (valor final)	262.833,3 por mm3 (RIC=71750)	40 % (64)	262333 (RIC: 72625)	267250 (RIC: 58125)
	Nitrógeno ureico inicial	15 mg/dl (RIC= 22,05)	27,5 % (44)	13 (RIC: 13,75)	38 (RIC: 55,3)
	Nitrógeno ureico final	20,5 mg/dL (RIC=28,67)	30,63 % (49)	13 (RIC: 22)	48,21 (RIC: 23,42)
	Sodio inicial	136,6 mEq/l (RIC=4,22)	38,75 % (62)	136,75 (RIC: 4,53)	135,95 (RIC: 3,88)
	Sodio final	136,6 mEq/L (RIC=5,56)	45 % (72)	136,13 (RIC: 4,7)	138,97 (RIC: 6,83)
	Potasio inicial	3,6 mEq/L (RIC=0,6)	37,5 % (37,5)	3,6 (RIC: 0,5)	3,6 (RIC: 0,83)
	Potasio final	3,44 mEq/L (DE=0,49)	40,63 % (65)	3,46 (DE=0,45)	3,38 (DE=0,64)
	Glucemia inicial	102 mg/dl (RIC=27)	38,13 % (61)	98,65 (RIC: 20,75)	116 (RIC: 42,5)
	Glucemia final	105,35 mg/dl (DE=27,78)	90% (144)	105,78 (DE=30,44)	104,05 (DE=21,42)
	AST** inicial	28 U/L (RIC=30,25)	25 % (40)	25 (RIC: 13,25)	100 (RIC: 136,5)
	AST** final	23,33 (RIC=25)	40,63 % (65)	20,5 (RIC: 12,25)	182 (RIC: 144)
	ALT** inicial	20.5 (RIC=22,75)	25 % (40)	20 (RIC: 12)	129 (RIC: 187,75)
	ALT** final	20 (RIC=32)	40,63 % (65)	17,5 (RIC: 21,63)	187,67 (RIC: 151)
	Bilirrubina total inicial	1,1 mg/dl (RIC=0,7)	55,63 (89)	0,9 (RIC 0,75)	1,55 (RIC: 5,6)
	Bilirrubina total final	1,3 mg/dl (RIC=4,65)	73,13 (117)	1 (RIC 0,68)	7,5 (RIC: 4,8)
	Bilirrubina indirecta inicial	0,8 mg/dl (RIC=0,69)	54,38 (87)	0,7 (RIC: 0,7)	1 (RIC: 1,65)
	Bilirrubina indirecta final	0,92 mg/dl (RIC=1,4)	73,75 (118)	0,72 (RIC: 0,5)	2,52 (RIC: 2,53)
	Bilirrubina directa inicial	0,2 mg/dl (RIC=0,28)	54,38 (87)	0,2 (RIC: 0,17)	0,9 (RIC: 4,4)
	Bilirrubina directa final	0,3 mg/dl (RIC=3,47)	73,8 (118)	0,2 (RIC: 0,19)	5,17 (RIC: 3,38)
	Creatinina inicial	1,12 mg/dl (RIC=2,63)	19,38 (31)	0,93 (RIC: 0,65)	3,94 (RIC: 5,14)
	Creatinina final	1,25 mg/dl (RIC=3,3)	31,25 (50)	1,02 (RIC: 1,92)	4,8 (RIC: 3,22)

* Las medias se reportan con desviación estándar (DE) y las medianas, con rango intercuartílico (RIC)

** TA: tensión arterial

lpm: latidos por minuto; rpm: respiraciones por minuto; AST: aspartato aminotransferasa; ALT: alanina aminotransferasa

**Cuadro 2:** Relación de las características sociodemográficas, clínicas y complementarias de los pacientes intoxicados con paraquat con su condición final, departamentos de Nariño y Putumayo, Colombia, 2013-2018

Variables	Característica	Categorías	OR crudo (IC)	p	OR ajustado (IC)	p
Sociodemográficas	Sexo	Masculino	1,14 (0,55-2,38)	0,7		
		Femenino	0,87 (0,42-1,8)	0,7		
	Departamento	Nariño	1,11 (0,54-2,33)	0,77		
		Putumayo	0,9 (0,43-1,86)	0,77		
	Procedencia	Rural	1,35 (0,54-3,33)	0,52		
		Urbano	0,74 (0,3-1,86)	0,52		
	Escolaridad	Con estudios	0,34 (0,15-0,73)	0,005	6,43	0,07
		Otros	2,97 (1,37-6,44)	0,005	(0,86-48,18)	
	Ocupación	Desconocido	3,74 (1,75-8,03)	< 0,001		
		Agricultor	0,58 (0,23-1,44)	0,234	Datos perdidos en más del 20 %	
		Ama de casa	Ninguna falleció	0,001		
		Otros	1,02 (0,4-2,6)	0,973		
	Régimen de salud	Subsidiado	1,12 (0,42-3,02)	0,823		
		No subsidiado	0,89 (0,33-2,38)	0,823		
Clínicas (cualitativas)	Cantidad ingerida (gravedad)	Moderada	0,38 (0,17-0,85)	**0,016**	2,77	
		Grave	2,63 (1,17-5,87)	**0,016**	(0,37-20,98)	
	Compromiso	Digestivo	0,31 (0,13-0,76)	**0,008**	1,4 (0,1-18,8)	
		Sin compromiso	0,082 (0,02-0,36)	**< 0,001**	Indicador	
		Renal	4,85 (2,02-11,63)	**< 0,001**	10,3 (0,45-237,11)	
		Otros	9,21 (3,5-24,26)	**< 0,001**	9,5 (0,5-180,76)	
	Falla renal	Sin falla renal	0,14 (0,06-0,31)	**< 0,001**	1,4 (0,12-16,61)	0,788
		Con falla renal	7,2 (3,22-16,08)	**< 0,001**		
	Diálisis	No	0,15 (0,05-0,43)	**< 0,001**	1,07 (0,06-18,78)	0,962
	Vía de exposición	Sí Oral	6,63 (2,35-18,7) No estimable	**< 0,001** 0,23		
		Otra	Ninguno falleció	0,23		
	Causa	Intencional	0,64 (0,23-1,82)	0,58		
		No intencional	1,56 (0,55-4,35)	0,58		
	Tiempo entre ingestión y	Mayor de 24 horas	0,75 (0,36-1,61)	0,47		
	atención médica	Menor de 24 horas	1,33 (0,62-2,78)	0,47		
	**Característica**	**Prueba estadística**	**Valor estadístico**	**p**	**OR ajustado (IC)**	**p**
Clínicas (cuantitativas)	Tiempo de estancia*	U de Mann-Whitney	1.749,5	**0,026**	**0,124 (0,03-0,6)**	**0,009**
		Prueba mediana	1,918	0,166		
	TA** sistólica	U de Mann-Whitney	2159	0,673		
	TA** diastólica	U de Mann-Whitney	1980	0,234		
	TA** media	U de Mann-Whitney	2.041	0,37		
	Temperatura	U de Mann-Whitney	2.248,5	0,9		
	Frecuencia cardíaca*	U de Mann-Whitney	1.771	**0,032**	**35,65 (1,44-884,78)**	**0,029**
		Prueba mediana	2,25	0,134		
	Frecuencia respiratoria	U de Mann-Whitney	1.542	**0,001**	1,29 (0,85-1,96)	0,227
		Prueba mediana	6,61	**0,014**		
	Hemoglobina inicial	t de Student	0,339	0,735		
	Hemoglobina final	t de Student	-0,235	0,815		
	Hematocrito inicial	t de Student	0,402	0,688		
	Hematocrito final	t de Student	-0,072	0,943		
	Glóbulos blancos inicial*	U de Mann-Whitney	933,5	**0,005**	1,52 (0,57-4,05)	0,406
		Prueba mediana	2,514	0,113		
	Glóbulos blancos final	U de Mann-Whitney	284	<0,001	Datos perdidos en más	
		Prueba mediana	9,075	0,003	del 20 %	
	Diferencial de neutrófilos	U de Mann-Whitney	634	<0,001	1,04 (0,93-1,16)	0,481
	inicial	Prueba mediana	13,52	<0,001		
	Diferencial de neutrófilos final	U de Mann-Whitney	178	<0,001	Datos perdidos en más	
		Prueba mediana	16,88	<0,001	del 20 %	
	Plaquetas inicial (278.346,2)	t de Student	-0,453	0,651		
	Plaquetas final (262.833,3)	U de Mann-Whitney	568,5	0,482		
	Nitrógeno ureico inicial	U de Mann-Whitney	412	**<0,001**	Datos perdidos en más del 20 %	
	Nitrógeno ureico final	Prueba mediana	25,968	**<0,001**		
		U de Mann-Whitney	214	**<0,001**	Datos perdidos en más del 20 %	
		Prueba mediana	22,438	**<0,001**		
	Sodio inicial	U de Mann-Whitney	774	0,6		
	Sodio final	U de Mann-Whitney	419	0,9		
	Potasio inicial	U de Mann-Whitney	823,5	0,773		
	Potasio final	t de Student (Welch)	0,538	0,596		
	Glucemia inicial	U de Mann-Whitney	23	0,953		
	Glucemia final	t de Student	0,104	0,918		
	AST** inicial	U de Mann-Whitney	150	**<0,001**	Datos perdidos en más del 20 %	
		Prueba mediana	30,17	**<0,001**		
	AST** final	U de Mann-Whitney	31,5	**<0,001**	Datos perdidos en más del 20 %	
		Prueba mediana	15,87	**<0,001**		
	ALT** inicial	U de Mann-Whitney	393,5	**<,0,001**	Datos perdidos en más del 20 %	
		Prueba mediana	14,19	**<0,001**		
	ALT** final	U de Mann-Whitney	69,5	**<0,001**	Datos perdidos en más del 20 %	
		Prueba mediana	16,60	**<0,001**		
	Bilirrubina total inicial	U de Mann-Whitney	259,5	**0,004**	Datos perdidos en más del 20 %	
		Prueba mediana	2,11	0,146		
	Bilirrubina total final	U de Mann-Whitney	17,5	**<,0,001**	Datos perdidos en más del 20 %	
		Prueba mediana	12,86	**<0,001**		
	Bilirrubina indirecta inicial	U de Mann-Whitney	377	0,058		
	Bilirrubina indirecta final	U de Mann-Whitney	31,5	**<0,001**	Datos perdidos en más del 20 %	
		Prueba mediana	10,631	**0,001**		
	Bilirrubina directa inicial	U de Mann-Whitney	167	**<0,001**	Datos perdidos en más del 20 %	
		Prueba mediana	12,68	**<0,001**		
	Bilirrubina directa final	U de Mann-Whitney	16	**<0,001**	Datos perdidos en más del 20 %	
		Prueba mediana	9,518	0,002		
	Creatinina inicial	U de Mann-Whitney	343	**<0,001**	1,73 (1,23-2,44)	
		Prueba mediana	30,594	**<0,001**		
	Creatinina fnal	U de Mann-Whitney	199	**<0,001**	Datos perdidos en más del 20 %	0,002
		Prueba mediana	17,661	**<0,001**		

* Se incluyen las variables categorizadas (ordinales) en el análisis multivariado

** TA: tensión arterial

lpm: latidos por minuto; rpm: respiraciones por minuto; AST: aspartato aminotransferasa; ALT: alanina aminotransferasa

Se resaltan en negrilla los valores de p menores a 0,05.

La mediana de edad de los pacientes fue de 22,5 años (rango intercuartílico, RIC: -9,75 años), con una distribución no gaussiana (Kolmogórov Smirnow: 0,192716; p<0,001); aproximadamente el 76 % de los pacientes tenía edades entre los 14 y los 30 años. La mediana de edad de los pacientes supervivientes fue de 23 años (RIC=10) y la de los fallecidos, de 21 (RIC=10,75), sin diferencia evidente entre estas (U de Mann-Whitney de 2,023 y p=0,26; prueba de la mediana: 2,65 y p=0,1).

**Figura 2:**
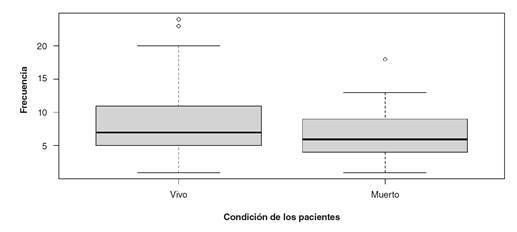
Distribución de los días de estancia hospitalaria según la condición final de los pacientes intoxicados por paraquat en un hospital de tercer nivel de complejidad en Pasto, Colombia, 2013-2018

En cuanto a las características clínicas, en el 39,37 % (n=63) de los casos no se supo la cantidad de paraquat ingerida y, de este porcentaje, el 26,98 % de los pacientes falleció (n=17); en uno de los casos no se conoció la condición final del paciente. Cerca del 75 % de ellos permaneció hospitalizado entre 1 y 10 días. La tasa de letalidad fue de 23,8 % (n=38). La figura 2 muestra la distribución de los días de estancia por condición final de los pacientes.

Las mujeres gestantes representaron el 7,1 % de los casos; su edad promedio era de 19,5 años y la mayoría tenía más de 20 semanas de gestación; en todos estos casos, la intoxicación se debió a la ingestión voluntaria con finalidad suicida.

Después del análisis ajustado, se determinó que el tiempo de estancia hospitalaria, la frecuencia cardiaca y la creatinina inicial, fueron las características relacionadas con la condición final de los pacientes intoxicados con paraquat.

## Discusión

En el estudio se encontró que la mayoría de los pacientes era de sexo masculino, con edades comprendidas en las tres primeras décadas de la vida, procedentes de áreas rurales de Nariño, pertenecientes al régimen subsidiado de salud y con algún grado de escolaridad. Estos hallazgos coinciden con los de otros trabajos ^12^^,^^25^^,^^32^^-^^34^.

Según la OMS, cerca de 703.000 personas se suicidan cada año ingiriendo paraquat, lo cual es la cuarta causa de muerte en el grupo etario de 15 a 29 años; el 77 % de estos eventos fatales sucede en países de bajos y medianos ingresos, y la mayoría de ellos tiene lugar en zonas rurales agrícolas ^24^. A pesar de que en el presente estudio se reportaron cifras de intoxicación más elevadas en la zona rural, posiblemente debido a las condiciones de vida deficientes y al impacto significativo del conflicto armado entre los agricultores, también la pobreza y el desempleo se asocian con las ideas y el comportamiento suicidas en estas poblaciones ^25^.

Asimismo, la mayor parte de los pacientes ingirieron la sustancia con fines suicidas, lo que concuerda con el hecho de que alrededor de un 20 % de todos los suicidios se cometen por intoxicación con plaguicidas ^24^^,^^32^.

En cuanto a las características clínicas, se determinó que una gran parte de los pacientes presentaba intoxicaciones moderadas, con compromiso digestivo y sin falla renal, y no requirieron diálisis. Proporcionalmente, la vía de intoxicación más frecuente fue la oral, sin que se encontraran diferencias entre esta circunstancia, o entre los signos vitales (salvo la frecuencia cardiaca) al comparar por condición final de los pacientes, hecho que no coincide con lo reportado en otros estudios, los cuales informan que la mayoría de los pacientes intoxicados presenta compromiso renal, pulmonar, metabólico y neurológico. Probablemente, ello se deba a que los pacientes ingresaron tardíamente a los servicios de salud de tercer nivel, a que el tiempo de la toma de los exámenes de laboratorio para el seguimiento no está estandarizado, y a que este tipo de productos adulterados se vende en la zona fronteriza con Ecuador ^35^^-^^37^.

En un estudio reciente en Antioquia, se reportó que la mayoría de los pacientes intoxicados con paraquat presentaba una afectación leve, discordancia que se debería a las diferencias regionales y a los diferentes tamaños de la población en cada estudio ^34^. Otra consideración en este sentido es que la mayoría de los estudios describe los compromisos orgánicos que conducen al deceso del paciente, mas no los efectos locales del contacto con el herbicida.

En cuanto a la distancia geográfica, la mayoría de los casos arribaron al hospital de tercer nivel más de 24 horas después de la exposición, lo que incide en su manejo terapéutico y el resultado clínico ^38^. Cabe anotar que no se encontraron artículos recientes que reporten la relación entre el tiempo de atención y la letalidad.

Se debe considerar que tres variables son categorizadas de modo ordinal para su análisis dado que presentan diferencias entre grupos (mas no en sus medianas). La primera fue el tiempo de estancia hospitalaria, categorizada con base en la experiencia clínica de la autora principal, quien, además del análisis estadístico de la distribución (figura 2), ha observado que los pacientes con evolución clínica y resultados complementarios desfavorables entre el quinto y el noveno día, tienen mayor riesgo de morir ^39^. La segunda fue la frecuencia cardíaca, clasificada como baja (bradicardia), normal o alta (taquicardia) ^40^. La tercera fue el número inicial de glóbulos blancos, tomando el valor mínimo como nulo (únicamente un paciente vivo y un paciente fallecido presentaron leucopenia, es decir, un conteo inferior a 4.000 células/mm^3^); el conteo normal se consideró hasta 11.000 por mm3; un valor de 16.000 por mm^3^ como punto de corte, basado en la mediana de los pacientes fallecidos (16.050 por mm3en este estudio) y que, además, es próximo al de 15.500 por mm^3^ reportado en un estudio en Irán; y, por último, uno de 20.000 por mm^3^, considerado en un estudio asiático como indicativo de mal pronóstico en pacientes intoxicados con paraquat ^8^^,^^41^^,^^42^.

A propósito de la mortalidad de pacientes intoxicados con paraquat, en el análisis ajustado de esta investigación se encontró que la muerte se relacionaba con características como tiempo de hospitalización, frecuencia cardiaca y creatinina en el momento del ingreso. Estudios en otras regiones indican como predictores de mal pronóstico a la cantidad ingerida, un nivel alto de creatinina, hiperamilasemia, hiperglucemia, niveles elevados de lactato y compromiso multiorgánico ^26^^-^^30^^,^^43^^,^^44^. Lee, *et al*., afirman, en detalle, que el 23,75 % de los pacientes fallecen después de la intoxicación por paraquat al referir factores como la cantidad ingerida, las condiciones clínicas y la alteración de los parámetros de laboratorio, entre otros ^45^.

Esto concuerda con los resultados expuestos por Xu, *et al*., quienes sostienen que la función renal anormal en el momento de la admisión y la lesión renal aguda son factores independientes de mortalidad ^46^. En contraste, en el presente estudio, fueron el tiempo de hospitalización y la frecuencia cardiaca los que se relacionaron con la mortalidad, lo que no tiene precedentes en publicaciones previas. Según algunos autores, otros factores de mal pronóstico en el paciente intoxicado con paraquat son la elevación de los niveles de glucemia y AST ^8^^,^^47^; no obstante, en este estudio, ni el nivel de la glucemia ni la elevación de las transaminasas (ni los resultados de ningún otro examen complementario, con excepción de la creatinina inicial), se asociaron con la condición final del paciente.

Un grupo especial afectado fue el de las mujeres gestantes, lo que coincide con otras publicaciones previas: Trakulsrichai, *et al*., describen que el 94,4 % de las pacientes ingirieron el paraquat intencionalmente, que la edad media fue de 22,7 años y la edad gestacional, de 23,1 semanas ^48^. Un vacío en la investigación de las intoxicaciones por paraquat es el referente a los efectos del bipiridilo en los fetos, lo que requiere estudios de seguimiento en los supervivientes.

Se encontraron varias publicaciones en la región de Antioquia referentes a la intoxicación con paraquat ^34^^,^^49^^,^^50^. En un estudio de cohorte retrospectivo de 67 casos durante siete años, se reportaron como factores relacionados con la mortalidad: dosis ingerida, carácter intencional de la ingestión, edad, ser hombre y elevación de algunos parámetros de laboratorios ^34^. Sin embargo, en el análisis de supervivencia y las comparaciones de grupos, no se consideró a toda la cohorte, sino solo a los pacientes fallecidos. Pese a su diseño transversal, en nuestro estudio se consideraron todos los casos en las comparaciones de grupos y el test de normalidad de Kolmogórov-Smirnov para variables cuantitativas (a diferencia del estudio en Antioquia, que utilizó el test de Shapiro-Wilk en una población mayor de 50 casos) ^34^.

Entre las limitaciones del presente estudio, se encuentran las relacionadas con el tipo de estudio, el cual no permite establecer la secuencia de los acontecimientos (exposición - enfermedad), ni la relación causal. También, hubo sesgo de confusión en cuanto a los antecedentes de los pacientes, pues no se contó con esta información en los registros, así como sesgo de información en todas las variables de laboratorio debido a la migración de los datos de una plataforma institucional a otra. Otra anotación importante es el subregistro de este tipo de eventos, lo que ocasiona pérdida de datos (como los de gases arteriales de control o registros regulares estandarizados de exámenes paraclínicos durante la estancia hospitalaria) y limita el análisis multivariado ^51^^,^^52^. Estas limitaciones no permitieron el análisis de los resultados de los exámenes de laboratorio finales ni de la cantidad de tóxico ingerido por los pacientes y, por lo tanto, tampoco de la variación de los resultados de los exámenes complementarios. En el estudio no se aplicaron escalas pronósticas, pero sí se evaluó su evolución con ayudas diagnósticas.

Para concluir, la proporción de letalidad entre los pacientes del estudio fue mayor en aquellos con estancias hospitalarias cortas (1 a 5 días y 6 a 10 días), frecuencias cardiacas elevadas y niveles elevados de creatinina al ingreso. El estudio puede ser útil como fundamento para la elaboración de una escala de gravedad que permita detectar a los pacientes de forma temprana e intervenir oportunamente.
